# Tumor Angiogenesis: Pericytes and Maturation Are Not to Be Ignored

**DOI:** 10.1155/2012/261750

**Published:** 2011-10-09

**Authors:** Elham Fakhrejahani, Masakazu Toi

**Affiliations:** Breast Surgery Department, Graduate School of Medicine, Kyoto University, Kyoto 606-8507, Japan

## Abstract

Angiogenesis, an essential component of tumor growth and survival, is regulated by complex interactions between several cell types and soluble mediators. Heterogeneous tumor vasculature originates from the collective effect of the nature of carcinoma and the complexity of the angiogenic network. Although the application of angiogenesis inhibitors in some types of cancers has shown clinical benefits, predictive markers to assess treatment effects have yet to be established. In this review, we focus on tumor vessel maturity as a potential marker for evaluating treatment response.

## 1. Introduction

Like normal tissues, malignant tissues are dependent on an adequate blood supply. Unlike normal tissues, however, angiogenesis is reactivated under pathological conditions, such as wound healing and malignancies [1]. Inflammation, metabolic stress and hypoxia are three major conditions involved in angiogenesis [2]. As malignant cells grow, the “demand” for nutrients and oxygen necessitates new “supply” routes, that is, new blood vessels. Early studies in this field have revealed that a tumor mass cannot exceed 1 mm^3^ without angiogenesis [3]. Although endothelial cells (ECs) are typically quiescent in humans [4], they can proliferate once the angiogenic switch turns on. This switch is off or differentially regulated in normal tissues based on the equilibrium between positive and negative angiogenic regulators. Upon receiving dominant proangiogenic stimuli from malignant cells or the tumor microenvironment through several effectors, such as vascular endothelial growth factors (VEGFs), platelet-derived growth factor (PDGF), placenta-derived growth factor (PlGF), hypoxia-inducible factor-1 (HIF-1*α*), angiopoietin-2, transforming growth factor *β* (TGF-*β*), or interleukin-8, ECs from preexisting vessels become activated. Activated ECs modify their interaction with perivascular cells (pericytes, PCs) and release proteases to degrade the surrounding basement membrane and extracellular matrix to facilitate EC proliferation and sprouting into the matrix [5]. Endothelial precursor cells (EPCs) from bone marrow also integrate with these growing vessels [6].

## 2. Tumor Vessel Maturity

Sprouting microvessels establish a plexus that keeps all tumor cells within a distance of 100–200 *μ*m from the blood supply [7]. This new architecture is considered “immature” and differs from normal (or mature) vascular structures in many ways. Immature vessels lack vascular organization and hierarchy and are unevenly distributed in tumor tissue. They are usually irregularly shaped, tortuous and dilated. As a result of impaired cell-to-cell attachment, abnormal basement membranes, and increased permeability, the new microvessels are leaky and cannot sustain balanced intravascular pressure gradients, which can lead to interstitial hypertension. ECs in the tumor vasculature are dependent on cell survival factors (e.g., VEGF) for survival in contrast to ECs in normal tissues [8]. Pericytes, which stabilize ECs and mediate EC survival and maturation in normal vasculature both through direct cell contact with ECs and paracrine signaling, are also abnormal in the tumor vasculature [9]. Pericytes are usually absent in the tumor vasculature or have loose associations with ECs, leaving most of the tumor microvessels immature. The basement membrane is rich in collagen type IV and a source of growth factors for ECs and PCs in normal vessels. In tumor vessels, the basement membrane has defects in its structure and composition and can provide a productive environment for metastatic malignant epithelial cells and proliferating ECs [10]. 

ECs are proliferative in a diverse manner based on immunostaining for proliferation molecules in cancer tissues [11].

**Figure 1:**
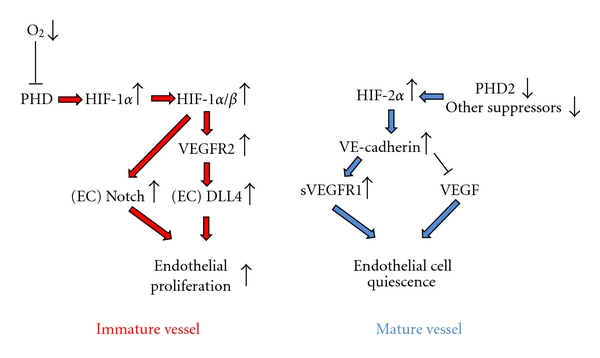
Hypoxia inducible factors (HIFs) and their role in endothelial cell proliferation. HIF-1*α* is hydroxylated by prolyl hydroxylase domain proteins (PHDs) and degraded in proteasomes under oxygenated conditions. When the oxygen level decreases, PHD activity is reduced, which leads to the accumulation of HIF-1*α*. Upon formation, the HIF-1*α*/*β* complex activates the transcription of numerous genes. Hypoxia and HIF-1*α* enhance the expression of VEGFR2, which induces DLL4 expression in the tip cell. Furthermore, DLL4 interacts with the Notch intracellular domain and increases its activity, which increases endothelial cell proliferation. Upregulation of HIF-2*α* due to lower degradation activates the junctional protein vascular endothelial cadherin (VE cadherin). VE cadherin induces a normalized endothelial phenotype by inhibiting VEGF-driven proliferation and upregulating the soluble isoform of the VEGF-trap VEGFR1 [12].

During tumor growth, the tumor vasculature develops as the angiogenic switch is intermittently turned on and off [12]. The imbalance between tumor cell growth and vascular formation may often cause a collapse of tumor cells and the new vasculature. Hypoxic areas exist heterogeneously in the majority of human solid cancer tissues [13]. HIFs are a family of transcription factors that are activated in response to hypoxic tumor tissue in different ways (Figure 1).

## 3. Angiogenesis in Human Cancer

Breast cancer is the most common cancer among women and is studied as an angiogenic carcinoma due to the high expression levels of proangiogenic factors, such as VEGFs, HIF-1*α*, TGF-*β*, or thymidine phosphorylase (TP), in carcinoma cells [14]. Various components of the angiogenic pathway have been studied as prognostic and predictive factors in breast cancer. Distinct patterns of vascularity (using an EC marker) in ductal carcinoma *in situ *(DCIS) might be useful to identify patients who are at risk of relapse [15]. Bone marrow metastasis as a direct result of interactions between carcinoma cells and the vascular network has been shown to have a higher prevalence in breast cancer patients with high-grade vascular tumors [16]. Angiogenesis has been used to predict the likelihood of tumor response to adjuvant chemotherapy or hormone therapy. Heterogeneous characteristics of biological markers, such as estrogen and progesterone receptors (ER and PgR, resp.) as well as human epidermal growth factor receptor-2 (HER-2), define the clinical outcome of breast cancer patients. Patients with ER-positive tumors have been shown to have prolonged disease-free survival and an increased likelihood of tumor response to endocrine therapies [17]. After the introduction of trastuzumab, a humanized monoclonal antibody directed against the extracellular domain of ErbB [18], HER-2-targeted therapies has changed the disease course of originally aggressive type of HER-2-positive breast cancer [19]. Triple-negative breast cancer (ER-, PgR-, and HER-2-negative; TNBC) comprises a heterogeneous subgroup of tumors characterized by an aggressive clinical course and poor survival and is not amenable to hormone therapy or HER2-directed agents [20].

Breast cancer is classified into distinct molecular subtypes based on expression profiling using DNA microarrays [21]. These subtypes are luminal A, luminal B, (HER2)-overexpressing, normal breast tissue-like, basal-like and the more recently identified subtype claudin-low [22]. These subtypes respond differently to therapy and are associated with different outcomes. The shortest survival times have been observed in patients with the basal-like and HER2-overexpressing subtypes [23]. Highly proliferative tumors, including those that are negative for the estrogen, progesterone, and HER2/neu receptors, have enhanced angiogenesis, which supports rapid growth and early metastases and have been found to have high levels of VEGF [24]. Many of the molecules involved in neovascularization pathways have become targets for antiangiogenic drugs, which are under evaluation in clinical trials or are currently administered in clinics [25]. Although many of these therapies, such as bevacizumab (anti-VEGF-A), are being used in combination with other therapeutic modalities, such as chemotherapy and endocrine therapy [26], studies are ongoing to find the optimal method to elucidate tumor response and overcome therapeutic resistance to antiangiogenic treatments. Genomic activation of VEGF-A is higher in TNBCs compared to other subgroups of breast cancer and suggests a specific role for bevacizumab treatment in this subgroup [27].

Although several distinguishing molecular features have been characterized in basal-like cancer cells, the microenvironmental features of this type of cancer have not been well characterized. A recent study on cocultures of breast cancer cell lines with fibroblasts has identified stromal interactions that distinguish basal-like from luminal-type breast cancers [28].

In a recent study on 1788 primary invasive breast cancers, VEGF expression is correlated with intrinsic subtypes with a higher frequency in luminal-type B, HER2, and basal-like types but not luminal-type A [24]. In another study of 564 tissue microarrays from primary tumors from premenopausal breast cancer patients who had been randomized to adjuvant tamoxifen or no adjuvant treatment, TNBCs show increased protein expression of epithelial growth factor receptor (EGFR) and VEGFR2, whereas the expression of VEGF-A is not a specific biomarker of TNBCs [27]. 

A pathological examination using CD34 of one thousand early-stage primary breast cancer specimens has shown that basal-like breast cancer and TNBCs had significantly higher microvessel densities (MVDs) than the nonbasal and non-TNBC groups [29]. In basal-like breast cancers, high MVD was associated with a larger tumor size and a higher grade. However, this association is not apparent in the TNBC group. CD34 has been reported to be present on endothelial progenitors [30]. Despite the fact that other morphological characteristics of the tumor vasculature are not available for evaluation, higher CD34-positive vascular structures might show a trend for the presence of immature vessels in basal-like breast cancers and TNBCs in this study. In addition, investigators have shown that in TNBCs and basal-like breast cancers, vascular invasion almost entirely consists of lymphatic vessels. Basal-like breast cancers have been reported to preferably metastasize to the brain and liver [31] and have been interpreted as distinct blood-borne metastases. However, the results from the afore-mentioned study might be explained by other molecular features of the interactions between basal-like malignant cells and endothelial cells.

In an *in vitro* study on breast cancer cell lines with different expression levels of ER*α*, Ang-1 mRNA and protein levels are higher in MDA-MB-231 cells (ER*α*-negative cell line) compared to those of MCF-7 cells, S30 cells, and HMEC (all ER*α*-positive cell lines) [32]. E2 treatment significantly attenuates Ang-1 mRNA and protein expression levels in S30 cells. Ang-1, VEGF, and CD31 staining in tumor samples from animals that have been inoculated with S30 and MDA-MB-231 cells reconfirms decreased angiogenesis *in vivo* in tumors that originated from the ER*α*-positive cell line.

Circulating tumor cells (CTCs) and disseminated tumor cells (DTCs) have a significant prognostic role in breast cancer patients [33]. These cells have heterogeneous biological characteristics that promote metastasis. In addition to the proangiogenic role of VEGF, VEGF stimulates tumor cell proliferation [34]. In a study of CTCs obtained from breast cancer patients, VEGF and its upstream regulators HIF-1 and phosphorylated-focal adhesion kinase (pFAK) are expressed in 73%, 56%, and 81% of detected CTCs, respectively [35]. Although the biological significance of these findings remains unknown, they implicate a possible role for angiogenic characteristics in the metastatic behavior of CTCs.

A genomic study on 134 primary breast cancers and 27 regional or distant metastases showed a high expression of a 13-gene cluster (VEGF profile) containing *VEGF*, *angiopoetin-like 4 *(*ANGPTL4*), and* adrenomedullin *(*ADM*) in tumors from patients with confirmed distant disease at the time of diagnosis (i.e., MetScore = 3) [36]. *VEGF* and *ANGPTL4* are endothelial cell growth inducers, and *ADM *is an inducer of lymphatic vessel growth [37, 38]. In addition, “perinecrotic” HIF-1*α* IHC staining correlates with the expression of the VEGF profile. Eight of these 13 genes had hypoxia response elements that are 2000 bp upstream of their start codons. The VEGF profile also correlates with the expression profile of three individual genes (*Snail, Twist, *and* HIF-1*α**) and the intrinsic subtype of breast cancer. 

BRCA-associated breast cancers are different from spontaneous breast cancers in many aspects, such as morphology, triple negativity, basal cytokeratin expression, and p53 mutations [39]. HIF-1*α* overexpression is more frequent in BRCA1-related breast cancer compared to that in sporadic cancer in a small series of 30 cases [40]. Elevated expression of HIF-1*α* and the loss of prolyl hydroxylase enzyme 3 (PHD3) and factor inhibiting HIF (FIH) in the nucleus have been observed in 125 BRCA-associated breast cancers [41]. PHD3 and FIH are responsible for the HIF-1*α* degradation and modulation observed in BRCA1-mutated breast cancers. This observation might explain how the BRCA1 tumors enhance hypoxic drive.

The number of microvessels that are positive for vasohibin-1 (a negative feedback regulator of angiogenesis) and vasohibin-1 mRNA levels in 17 breast ductal carcinomas *in situ* (DCIS) is significantly lower compared to those of 22 invasive ductal carcinomas [42]. This difference has not been observed when analyzing CD31. However, the number of vasohibin-1-positive microvessels and vasohibin-1 mRNA levels shows significant correlations with the Ki-67-labeling index and a high nuclear and histological grade in DCIS cases.

Multiple roles of COX-2 in tumor angiogenesis, such as VEGF production, the promotion of vascular sprouting, migration, and tube formation, have been well studied [43]. COX2 expression occurs in malignant cells and under preneoplastic conditions, such as esophageal dysplasia [44]. In a study of 49 DCIS samples without any invasive component, the investigators have shown that VEGF expression is significantly associated with COX-2 expression [45]. This result is in agreement with a xenograft model in a human DCIS study that observed that COX2 upregulation in DCIS xenografts increased VEGF and MMP14 expression [46].

## 4. Antiangiogenic Therapies and Pericytes

One of the main mechanisms of action of antiangiogenic agents is vascular normalization [47]. These drugs change the balance of pro- and antiangiogenic factors in the tumor tissue and fix the delivery system to ensure that oxygen and therapeutic drugs are effectively distributed to a larger number of tumor cells. In other words, they help the immature tumor vasculature mature. One of the well-studied factors that maintains vascular maturity is the close association between PCs and ECs [48]. Activated ECs sprout and form an endothelial tube, which is a lumen with an EC lining. These ECs stop proliferating and secrete PDGF to recruit PCs and progenitor PCs (e.g., from bone marrow), which express PDGF receptors (PDGF-Rs) [49]. Recruited PCs proliferate and encapsulate these new channels. Newly formed vessels that are enveloped with PCs mature and stop remodeling [50]. Pericytes stabilize the neovessels and are crucial for EC survival by locally releasing VEGF and angiopoietin-1 [51]. Therapies that target VEGF have been observed to selectively prune ECs that are not covered by PCs [52]. Paracrine EC-PC signaling that is mediated by members of the PDGF family may account for the relative resistance of more mature vessels to anti-VEGF therapies [53]. Although bevacizumab has shown clinical efficacy in the treatment of several tumor types, no treatment-predictive-markers have been established in clinics [54]. 

**Figure 2:**
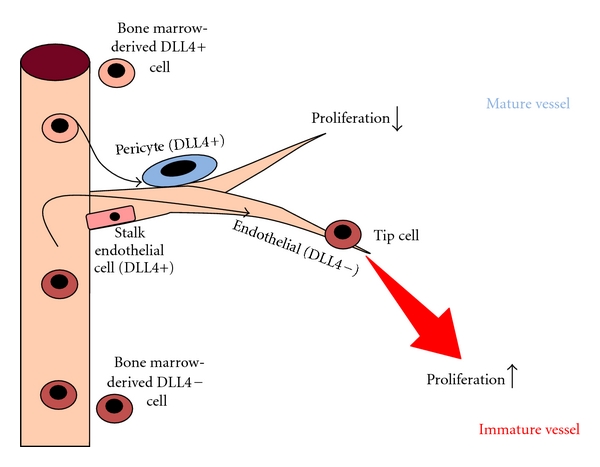
Delta-like ligand 4 (DLL4) and its expression in bone marrow-derived cells. The expression level of DLL4 dictates the role of bone marrow-derived cells in the neovasculature. DLL-4-Notch signaling controls tip cell versus stalk cell fate in endothelial cells and has a regulatory effect on pericyte formation [66].

Hypertension (HTN) has been reported to be a common event associated with bevacizumab treatment, possibly due to EC-derived nitric oxide reduction and consecutive vascular smooth muscle constriction, which increases vascular resistance [55]. In a large meta-analysis, the incidence of HTN in bevacizumab-treated cancer patients was 23.6% with 7.9% of patients with grade 3-4 of HTN [56]. Recent studies on metastatic colon cancer [57], renal cell carcinoma [58], lung cancer [59], and breast carcinoma [60] have shown an association between bevacizumab-related HTN and a better outcome, which suggests that HTN may be a predictive factor. An exploratory retrospective analysis of samples from the AVF2119 g phase III trial has shown no progression-free survival (PFS) benefits from the addition of bevacizumab to neoadjuvant capecitabine in metastatic breast cancer patients and has revealed that subgroups with low expression of endothelial neuropilin-1 (NRP1), TP, VEGF-C, or endothelial delta-like ligand 4 (DLL4) showed trends toward PFS benefits [61]. This result is compatible with the study on rectal carcinoma showing that bevacizumab upregulated NRP1 in tumor-associated-macrophages [62]. NRP1 is a co-receptor for VEGF and is expressed on ECs, tumor cells and vascular smooth muscle cells [63]. Preclinical studies have shown that combining bevacizumab treatment with anti-NRP1^B^ treatment resulted in additive effects to inhibit tumor growth [64]. DLL4 expression on ECs activates the Notch signaling pathway, which results in the regulation of tumor angiogenesis in a VEGF-independent manner [65]. DLL4 is expressed by ECs, EPCs, and bone marrow-derived *α*-smooth muscle actin (*α*-SMA)-positive mural cells (e.g., pericytes) (Figure 2) [66]. Disruption of DLL4 signaling in combination with anti-VEGF treatment has shown additive effects on tumor growth [67]. Compared to 64.5% of DLL4-negative vessels, 98.7% of DLL4-positive tumor vessels are surrounded by *α*-SMA-positive pericytes in bladder cancer [68].

Monocyte chemoattractant protein-1 (MCP-1) is a protein that is produced by breast cancer cells and stromal cells and participates in VEGF-mediated angiogenesis [69]. We have previously shown that MCP-1 alone or in combination with VEGF is a significant prognostic factor in breast cancer [69]. MCP-1 is a direct gene target for TGF-*β* in ECs and mediates the angiogenic effect of TGF-*β* by promoting the recruitment of mural cells to ECs [70]. Although the immunoregulatory role of MCP-1 in breast tumor metastasis has been shown [71], its involvement in tumor vessel maturation might be another factor in metastasis promotion.

**Figure 3:**
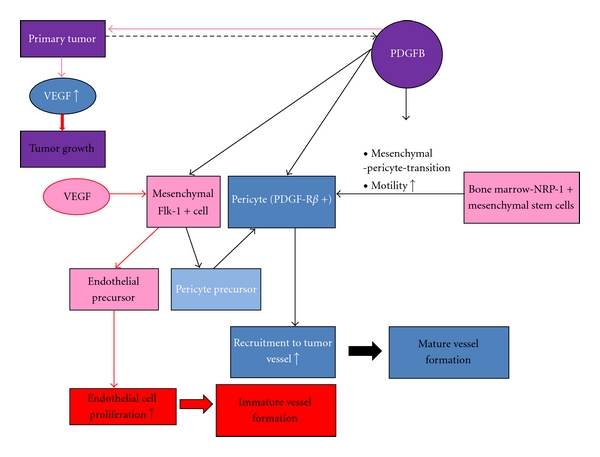
Tumor cell-derived PDGF-B induced pericyte differentiation. Incorporation of pericytes into newly formed vessels is one of the key steps to terminate angiogenesis. Studies in animal models have shown that PDGF-B is responsible for (1) differentiation of pericytes from mesenchymal stem cells through the PDGF-B-NRP-1 signaling pathway (2) increased recruitment and attachment of newly differentiated pericytes into newly formed tumor vessels. In addition, Flk1+ cells differentiate into endothelial cells or pericytes upon stimulation by VEGF or PDGF-B [72].

**Figure 4:**
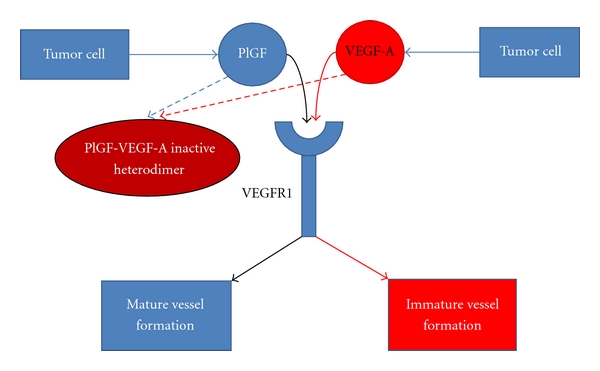
Negative regulation of angiogenesis by the PlGF-VEGFR1-mediated signaling pathway. In PlGF-producing mouse Lewis lung and human tumors, the tumor vasculature that is induced by PlGF-1 and -2 is covered by pericytes and is less leaky compared to the tumor vasculature that is induced by VEGF-A. This result might be caused by the formation of angiogenically inactive PlGF-VEGF heterodimers [74].

An *in vitro* study using mouse embryonic mesenchymal stem cells has shown that tumor cell-derived PDGF-B plays an important role in the differentiation and recruitment of PCs through NRP1 signaling (Figure 3) [72]. Less aggressive cell lines, such as MCF-7 cells (a noninvasive breast cancer cell line), have been shown to cause more recruitment and attachment of PCs to blood vessels compared to aggressive cell lines, such as MDA-MB-231. Another animal study has shown that tumor-derived PDGF-BB upregulates the transcription of stromal derived factor-1*α* (SDF-1*α*) in ECs [73]. EC-derived SDF-1*α* forms a chemotactic gradient that recruits CXCR4+ pericytes and smooth muscle progenitor cells during cancerous vascular remodeling. Another malignant cell-derived factor involved in the normalization of the tumor vasculature is PlGF [74]. This growth factor only binds to VEGFR1, forms a heterodimer with VEGF that inhibits angiogenesis, and leads to vascular remodeling by forming pericyte-enriched vascular networks (Figure 4).

The combination of anti-VEGF-R and anti-PDGF-R antibodies enforces tumor vessel regression by interfering with PC-mediated EC survival mechanisms [75]. Other studies have revealed that inhibiting PDGF-R improves tumor drug uptake in experimental tumor models [76]. Hepatocyte growth factor (HGF), a mesenchymal-derived protein, is a potent chemokine that regulates the growth and motility of many cell types, such as vascular smooth muscle cells [77]. Upon activation by angiopoietin-1, ECs produce HGF, which in turn leads to the migration of smooth muscle cells toward ECs [78]. XL880 (foretinib, GSK1363089) and XL184 (cabozantinib) are small molecule inhibitors that potently block multiple RTKs including VEGFR and the receptor of hepatocyte growth factor, c-Met. In a mouse model of pancreatic islet tumors, treatment with XL880 or XL184 led to rapid, widespread, and progressive regression of the tumor vasculature and reduced pericyte numbers [79].

## 5. Quantification of Angiogenesis

In clinical routines, the response to antitumor therapies (chemo- or radiotherapy) is usually assessed by measuring tumor size [80]. However, strategies with more specificity are needed to provide information about tumor vitality. Imaging of angiogenic and antiangiogenic behaviors is an essential component to evaluate antiangiogenic therapy. Various quantitative imaging techniques, such as positron emission tomography (PET) [81], magnetic resonance imaging (MRI) [82], or X-ray-computed tomography (CT) [83], are being used to evaluate changes in the tumor vasculature following the administration of angiogenesis inhibitors. However, these techniques can only reflect the physiological changes and require further development before they can be accepted as surrogate endpoints. These techniques require the use of blood-pool contrasting agents to characterize tissue blood volume, perfusion, and vessel permeability [84]. Although small vessels have been visualized using these methods in animal models [85], their clinical application is limited due to the long scanning time or experimentally constructed agents. Therefore, biopsy and histopathological evaluation are currently the accepted gold standards in clinical trials. MVD has been a well-studied marker to assess neoangiogenesis in several malignancies, such as breast carcinoma. Endothelial cells express different cell surface markers as a function of developmental age [86]. Differentiated endothelial cell specific markers (e.g., CD31 or CD34) are commonly used for tissue analysis by single immunohistochemical staining and MVD analysis. A histological examination of 512 breast cancer samples has shown that stromal PDGF-R expression significantly correlates with less favorable clinicopathological parameters (e.g., histopathological grade, estrogen receptor negativity, and high HER2 expression) and shorter survival rates [87]. 

**Figure 5:**
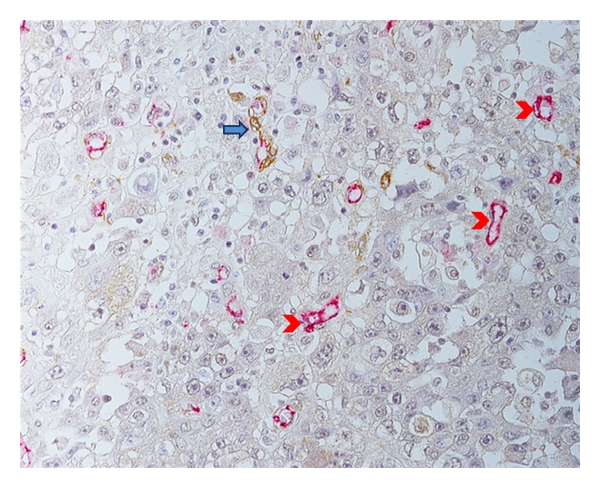
Double-immunostaining of pericytes and endothelial cells in breast cancer. Double immunostaining for CD31 (red) and *α*-SMA (brown) is shown. The blue arrow indicates a vessel that is CD31+/*α*-SMA+; the red arrowheads indicate vessels that are only CD31+. Magnification × 200.

Although the single immunohistochemical staining method allows the analysis of morphological aspects of angiogenesis, such as vessel size, shape, and density, these criteria might not be sufficient to assess vascular function. Double immunohistochemical staining using an EC marker and a PC marker (e.g., *α*-SMA) can be used to evaluate the morphology and the maturity of tumor microvessels by differentiating between the vessels with or without PCs. Our studies have shown that this method is easily applicable to tissue samples from formalin-fixed paraffin-embedded pathological archives (Figure 5). Our studies of predictive factors and prognostic values of microvessel maturity have used EC-PC double staining in primary breast cancer patients and are ongoing in patients who have undergone neoadjuvant chemotherapy. We believe that this new method has great potential to evaluate the prognostic and predictive role of angiogenesis from a new prospective. Brain metastases from nonsmall cell lung carcinomas have a higher proportion of mature vessels compared to primary tumors independent of the vascular pattern of the primary tumor [88]. This discordance between the vascular characteristics of primary and secondary cancers suggests that the maturity of microvessels in primary tumors should be considered when assessing angiogenesis as a prognostic or predictive factor. In another study of a small number of breast cancer samples (*n* = 50), blood vessel maturity was assessed by the positivity of LH39 at the lamina lucida of mature microvessels [89]. Mature blood vessels are defined by staining with antibodies to LH39 and CD31, using double immunohistochemistry, whereas immature blood vessels are characterized by positive CD31 staining. The vascular maturity index (VMI) is defined as the percentage of the fraction of mature vessels (LH39-positive)/total number of vessels (CD31-positive). TP expression but not VEGF expression is correlated with a low VMI showing intense vascular remodeling in TP-expressing cancers. TP is highly expressed in breast cancer cells and inflammatory cells in the stroma [90]. Its antiapoptotic and proangiogenic roles have been well studied. Therefore, the assessment of vessel maturity and microvessel count may identify patients who might benefit more from specific chemotherapy or antiangiogenic therapies.

Circulating endothelial cells (CECs) and circulating progenitor cells (CPCs) are novel surrogate markers of vascular disruption and repair in cancer [91]. In a study of 160 breast cancer patients, CECs are significantly higher in the poor prognostic group based on the Nottingham Prognostic Index (NPI), whereas CPCs are lower in the poor prognosis group [92]. Although vascular invasion and tumor size are independently associated with CECs, Her-2 status positively predicts CPCs. Circulating endothelial cell analysis in 46 advanced breast cancer patients treated with metronomic cyclophosphamide, capecitabine, and bevacizumab demonstrates that a high level of CECs indicates an active vascular turnover and predicts a prolonged clinical benefit for treatment, whereas low CEC counts are evident during tumor progression [93]. Decreased levels of CECs are accompanied by increased levels of VEGF-A and basic fibroblast growth factor, which suggests a switch toward a different type of cancer vascularization. High levels of CECs, which indicate active vascular remodeling, are shown to be associated with therapeutic responses. However, low CEC counts in cases of tumor progression might indicate a more stable microvasculature in these tumors (i.e., mature microvessels).

## 6. Conclusion

Combining different chemotherapeutic agents and angiogenic inhibitors normalizes the tumor vasculature, and the essential role of PCs in microvessel maturity and the concomitant histological evaluation of EC-PC interactions, and tumor microvessel morphology seems to be inevitable. In addition, the influence of lymphangiogenesis and EC interactions with tumor cells that express angiogenic receptors should also be investigated. Many new angiogenic inhibitors target pathways that are involved in the recruitment of pericytes to tumor microvessels. Therefore, it is essential to assess PCs in parallel with ECs when studying the tumor vasculature. This evaluation, which can be performed in a diagnostic pathology laboratory, can be used as a decision-making tool to select patients who might benefit from antiangiogenic therapies.

## Abbreviations

EC: Endothelial cell
PC: Pericyte
VEGF: Vascular endothelial growth factor
PDGF: Platelet-derived growth factor
HIF: Hypoxia-inducible factor
ER: Estrogen receptor
PgR: Progesterone receptor
HER-2: Human epidermal growth factor receptor-2
TNBC: Triple-negative breast cancer.
